# De Novo Immunoglobulin A Vasculitis Following Exposure to SARS-CoV-2 Immunization

**DOI:** 10.31486/toj.21.0083

**Published:** 2021

**Authors:** Muner M. B. Mohamed, Terrance J. Wickman, Agnes B. Fogo, Juan Carlos Q. Velez

**Affiliations:** ^1^ Department of Nephrology, Ochsner Clinic Foundation, New Orleans, LA; ^2^ Department of Pathology, Microbiology and Immunology, Vanderbilt Medical Center, Nashville, TN; ^3^ The University of Queensland Faculty of Medicine, Ochsner Clinical School, Brisbane, Queensland, Australia

**Keywords:** *BNT162b2 vaccine*, *COVID-19*, *glomerulonephritis*, *glomerulonephritis–IgA*, *purpura–Schonlein-Henoch*, *vaccines*

## Abstract

**Background:** Immunizations have been previously described as potential triggering events for the development of certain glomerular diseases. However, glomerular disease occurrences are being reported after exposure to a severe acute respiratory syndrome coronavirus 2 (SARS-CoV-2) vaccine.

**Case Report:** A 50-year-old male presented to a nephrology clinic for evaluation of persistent proteinuria. Six weeks prior to evaluation, the patient had reported developing a rash 2 weeks after receiving the first dose of a SARS-CoV-2 vaccine (BNT162b2 mRNA, Pfizer, Inc). His primary care provider treated the rash with corticosteroids, leading to partial improvement of the skin lesions. Three weeks after the first vaccine injection, the patient received his scheduled second vaccine injection. Within 2 days, the rash reappeared. This time, the lesions were more severe in nature. Skin biopsy revealed immunoglobulin A (IgA)-dominant leukocytoclastic vasculitis. After the patient completed 2 weeks of oral corticosteroids, urinalysis revealed proteinuria, and consultation with nephrology was requested. On examination, healing papules were noted on his legs. Serum creatinine 2 weeks after the second dose of vaccine was 0.9 mg/dL. Microscopic examination of the urinary sediment revealed acanthocytes. Urine protein to creatinine ratio 3 weeks after the second dose of vaccine was 1.1 g/day. Serum complements were normal, and all pertinent serology was negative. Kidney biopsy findings were consistent with IgA nephropathy.

**Conclusion:** The clinical presentation and pathologic findings in this case strongly suggest that the Pfizer SARS-CoV-2 vaccine can trigger a clinical syndrome compatible with Henoch-Schönlein purpura. The recurrence of the rash following the second dose argues for a definite causal association by the Naranjo criteria.

## INTRODUCTION

Individuals affected by coronavirus disease 2019 (COVID-19) may be at risk of acquiring certain forms of glomerular disease.^1,2^ Reports of acute glomerular syndromes have emerged since the widespread vaccination against infection by severe acute respiratory syndrome coronavirus 2 (SARS-CoV-2) occurred across the globe between January and June 2021.^3,4^ Because immunizations have been previously described to trigger certain forms of glomerular disease,^5-7^ the recent reports of such events occurring after COVID-19 vaccination are not entirely surprising. However, data are still scarce regarding what types of glomerular pathologies can be elicited by COVID-19 vaccines. Reports published in 2021 describe cases of immunoglobulin A (IgA) nephropathy relapsing after exposure to COVID-19 vaccination.^8-11^ However, to our knowledge, de novo IgA vasculitis in an adult following a COVID-19 vaccine has not been previously reported.

We describe the case of a 50-year-old male who developed dermatologic and renal manifestations of IgA vasculitis after receiving a SARS-CoV-2 vaccine.

## CASE REPORT

**Figure 1.**
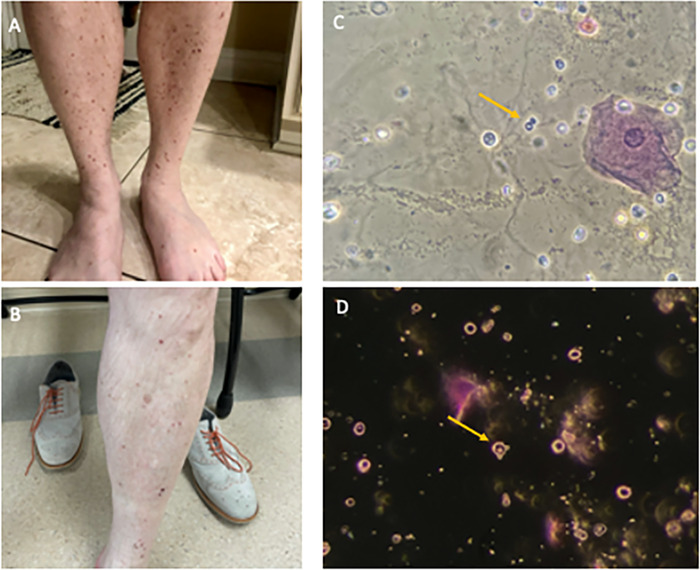
Clinical manifestations of immunoglobulin A vasculitis. (A) Palpable purpuric rash on the lower extremities 2 weeks after the patient received the first dose of the severe acute respiratory syndrome coronavirus 2 mRNA vaccine (BNT162b2, Pfizer, Inc) and (B) after 2 weeks of oral corticosteroids. Microscopic examination of urinary sediment stained with Sternheimer-Malbin stain revealed glomerular hematuria, characterized by acanthocytes (arrows) inspected under (C) phase-contrast microscopy and (D) dark-field microscopy illumination. Original magnification ×400.

A 50-year-old male presented to a nephrology clinic for evaluation of persistent proteinuria. His medical history was only pertinent for seasonal allergy and a mild COVID-19 infection that did not require hospital admission 4 months prior to evaluation. He was taking no medications. Six weeks prior to presentation, the patient had developed a skin rash on his lower legs that erupted 2 weeks after he received the first dose of a SARS-CoV-2 mRNA vaccine (BNT162b2 (mRNA), Pfizer, Inc) (Figure 1). His primary care provider treated the rash with fluocinonide 0.05% cream, applied to the affected area twice daily for 1 to 2 weeks. Three weeks after the first vaccine injection, the patient received his scheduled second vaccine injection. Within 2 days, the skin rash reappeared. This time, the lesions were more severe in nature, with violaceous nonblanching papules and blisters involving the same area on his lower legs but also affecting his thighs, the dorsal aspect of his forearms, lower abdomen, upper back, and buttocks. He reported concomitant myalgias. He denied gross hematuria or dark or foamy urine. Skin biopsy revealed IgA-dominant leukocytoclastic vasculitis. The patient was treated with prednisone for 3 weeks: 60 mg daily for the first week, 40 mg daily for the second week, and 20 mg daily for the third week.

After completion of 2 weeks of oral corticosteroids, urinalysis revealed proteinuria, and a nephrology consultation was requested. On examination in the nephrology clinic, the patient's vital signs were normal with blood pressure of 122/81 mmHg. Healing papules were noted on his lower extremities, but otherwise the physical examination was normal. Laboratory data are shown in Table 1. Two weeks after the second dose of vaccine, serum creatinine was normal at 0.9 mg/dL. Microscopic examination of the urinary sediment revealed acanthocytes (Figure 1). Three weeks after the second dose of vaccine, urine protein to creatinine ratio (UPCR) was 1.1 g/day. Serum complements were normal, and all pertinent serology was negative. A second UPCR repeated 1 week later (4 weeks after the second dose of vaccine) was still elevated at 0.8 g/day. Microscopic examination of the urinary sediment was repeated, and acanthocytes were again identified.

**Table 1.** Patient's Laboratory Data Before and After Vaccination

Parameter	Reference Range	4 Days Before First Dose of Vaccine	2 Weeks After Second Dose of Vaccine	3 Weeks After Second Dose of Vaccine^a^
**Clinical chemistry**				
Sodium, mmol/L	136–145	138	140	
Potassium, mmol/L	3.5–5.1	4.4	4.2	
Chloride, mmol/L	95–110	104	103	
Bicarbonate, mmol/L	23–29	26	27	
Anion gap, mmol/L	5–15	8	10	
Blood urea nitrogen, mg/dL	6–20	15	11	
Creatinine, mg/dL	0.5–1.4	0.9	0.9	
eGFR, mL/min/1.73 m^2^	>60	>60	>60	
Calcium, mg/dL	8.7–10.5	9.3	9.8	
Glucose, mg/dL	70–110	107	103	
Alkaline phosphatase, U/L	55–135	149	135	
Protein total, g/dL	6.0–8.4	7.5	8.3	
Albumin, g/dL	3.5–5.2	3.8	3.4	
Bilirubin total, mg/dL	0.1–1.0	0.4	0.7	
Aspartate transaminase, U/L	10–40	22	13	
Alanine transaminase, U/L	10–44	28	25	
C-reactive protein, mg/L	0.0–8.2		100	
Erythrocyte sedimentation rate, mm/h	0–10		92	
**Complete blood count**				
Hemoglobin, g/dL	14.0–18.0	14.3	13.3	
Platelets, K/uL	150–350	339	407	
White cell count, K/uL	3.90–12.70	9.4	9.2	
Neutrophils, %	38.0–73.0	69.8	67.7	
Lymphocytes, %	18.0–48.0	20.7	22.3	
Monocytes, %	4.0–15.0	6.6	7.1	
Eosinophils, %	0.0–8.0	0.2	0.2	
Basophils, %	0.0–1.9	0.04	0.02	
**Immunology**				
ANA screen	Negative <1:80			Negative <1:80
Complement (C3), mg/dL	50–180	N/R	N/R	148
Complement (C4), mg/dL	11–44	N/R	N/R	25
Antistreptolysin O titer, IU/mL	<200		113	
C-ANCA	<1:20 titer	<1:20 titer		
P-ANCA	<1:20 titer	<1:20 titer		
**Infectious disease**				
Hepatitis B surface antigen		Negative		
**Urine**				
Color	Yellow, straw, amber	Yellow		Yellow
Appearance	Clear	Cloudy		Hazy
Specific gravity	1.005–1.030	≥1.030		1.025
pH	5.0–8.0	5.0		5.0
Glucose	Negative	Negative		Negative
Protein	Negative	Negative		2+
Ketones	Negative	Negative		Negative
Occult blood	Negative	Negative		2+
Nitrite	Negative	Negative		Negative
Bilirubin	Negative	Negative		Negative
Leukocytes, hpf	0–4	Trace		Trace
Red blood cells, hpf	0–5			10
White blood cells, hpf	None-Occ	3		18
Bacteria		Rare		None
Protein to creatinine ratio, g/day	0.00–0.20			1.1

^a^Clinical chemistry and complete blood count were not done 3 weeks after the second dose of vaccine.

ANA, antinuclear antibody; C-ANCA, cytoplasmic-antineutrophil cytoplasmic antibodies; eGFR, estimated glomerular filtration rate; N/R, not reported; P-ANCA, perinuclear-antineutrophil cytoplasmic antibodies.

**Figure 2.**
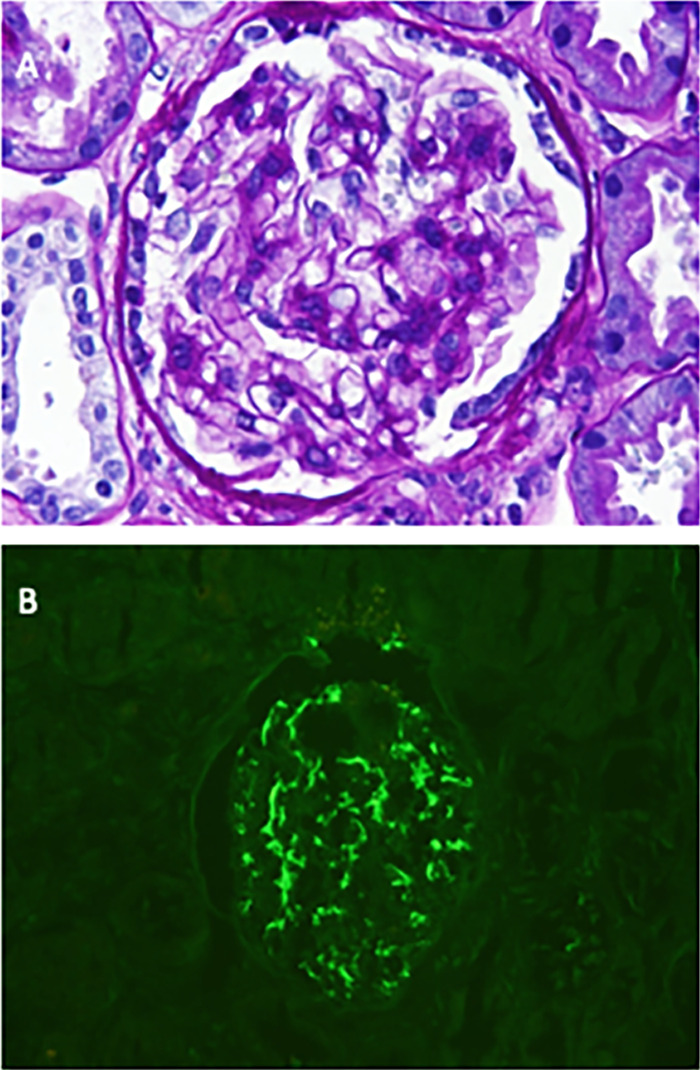
Kidney biopsy specimen showing pathologic features of immunoglobulin A (IgA) nephropathy. (A) Focal mild mesangial hypercellularity was present (periodic acid Schiff stain, original magnification ×400). (B) IgA-dominant granular diffuse global mesangial staining for IgA was present, 3+ on a 0 to 3+ scale (anti-IgA immunofluorescence, original magnification ×400).

Upon discussion of risks and benefits of establishing a diagnosis, the patient agreed to a kidney biopsy. The biopsy was performed without complications and consisted of 16 glomeruli by light and immunofluorescence microscopy with 2 globally sclerosed with diagnostic changes of an IgA nephropathy with dominant polyclonal, lambda slightly more than kappa, mesangial staining by immunofluorescence microscopy, and corresponding scattered mesangial deposits by electron microscopy. Mild mesangial hypercellularity was present in only 1 of the 16 glomeruli in the light microscopy sample, with no endocapillary hypercellularity, no segmental sclerosis, minimal (approximately 5%) interstitial fibrosis and tubular atrophy, and no crescents or necrosis (Figure 2). The findings were diagnosed as IgA nephropathy, which in the context of the skin lesions was consistent with IgA vasculitis. Although the MEST-C score has not been validated in IgA vasculitis, these lesions correspond to M0E0S0T0C0 (M=mesangial proliferation, E=endocapillary proliferation, S=segmental sclerosis, T=tubulointerstitial fibrosis, C=crescents).

**Figure 3.**
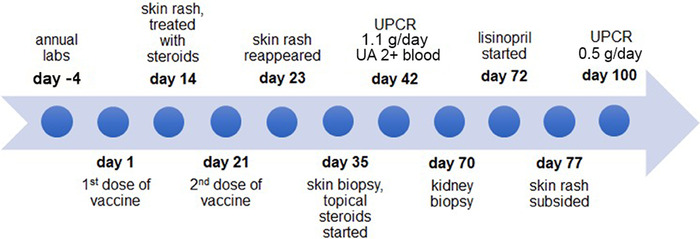
**Timeline of case report. Day –4 corresponds to 4 days before the patient received the first dose of vaccine.** UA, urinalysis; UPCR, urine protein to creatinine ratio.

After the kidney biopsy, the patient began treatment with lisinopril 10 mg orally daily. No further immunosuppression was given. At a follow-up visit 1 week after the kidney biopsy, the rash had subsided completely. The UPCR 4 weeks after starting the lisinopril was 0.5 g/day. Figure 3 presents a timeline of the case.

## DISCUSSION

Emergence of glomerular disease following vaccination against viral infections is a known phenomenon. A T lymphocyte–mediated cellular response appears to be a mechanistic link between the development of antibodies against a virus and the pathogenesis of a glomerular lesion.^12,13^ Perhaps the glomerular entity best characterized as being associated with immunizations is minimal change disease. Several reports demonstrate that diffuse podocyte effacement and nephrotic syndrome can occur following vaccination for influenza and other viruses.^5,6,14-19^ The COVID-19 pandemic resulted in an unprecedented volume of vaccinations worldwide. Not surprisingly, some reports of glomerular disease development^20,21^ or relapse of a preexisting glomerular disease have been published, including some describing IgA nephropathy.^8-11^ In our case, the clinical presentation and pathologic findings strongly suggest that the SARS-CoV-2 BNT162b2 (mRNA) vaccine from Pfizer can trigger a clinical syndrome compatible with IgA vasculitis (Henoch-Schönlein purpura)*.* The timing of the initial appearance of the rash with respect to the vaccination and the recurrence of the rash following reexposure to the vaccine argue for a “definite” causal association by the Naranjo criteria.^22^

IgA vasculitis can be triggered by many factors. IgA vasculitis occurrences after immunizations have been reported in children. A case-control study in children found an increased risk of IgA vasculitis within 12 weeks after administration of the measles, mumps, and rubella vaccination (odds ratio 3.4).^23^ The flu vaccine has been reported to be the vaccine type most commonly associated with vasculitis.^24^ Cutaneous manifestations after a COVID-19 vaccine have been reported. In a registry-based study of 414 patients who received mRNA COVID-19 vaccines, delayed large local reactions were most common.^25^ A case reporting Henoch-Schönlein purpura without additional organ involvement after a BNT162b2 (mRNA) vaccine was published in July 2021,^26^ making our case, to our knowledge, the first de novo Henoch-Schönlein purpura case with both a cutaneous manifestation and kidney involvement.

Published reports describe glomerular diseases after COVID-19 vaccines, including a case of antineutrophil cytoplasmic antibody vasculitis,^21^ granulomatous vasculitis,^27^ and 7 cases of relapse of IgA nephropathy following exposure to COVID-19 vaccines (Table 2).^8-11^

**Table 2.** Patient Demographics and Clinical Characteristics of Previously Reported Cases of Post-COVID-19 Vaccine Immunoglobulin A Nephropathy (IgAN)

Study	Age, Sex, Race	SARS-CoV-2 Vaccine	Time of Flare-Up After COVID-19 Vaccine	Year IgAN First Diagnosed	Treatment	Gross Hematuria Events During Disease Course	Persistent Microscopic Hematuria	Proteinuria in 2020, g/d	Proteinuria Between SARS-CoV-2 Vaccine Doses, g/d	Proteinuria after Last SARS-CoV-2 Vaccine Dose, g/d
Rahim et al, 2021^8^	52, F, Asian	BNT162b2 (Pfizer)	24 hours after the second dose	2017	RAASi	Yes	N/R	633.1 mg/g^a^	N/R	2,411 mg/g after 48 hours, 1,441 mg/g after 5 days^a^
Negrea and Rovin, 2021^9^	38, F, W	mRNA-1273 (Moderna)	8 to 24 hours after the second dose	2005	RAASi	At presentation; during 1 episode of gastroenteritis; occasionally after yearly influenza vaccine	Yes	0.63	0.82	1.40
	38, F, W	mRNA-1273 (Moderna)	8 to 24 hours after the second dose	2019	Cyc + pred for 6 months, followed by RAASi	At presentation only	Yes	0.43	0.59	0.4
Perrin et al, 2021^10^	22, M, N/R	mRNA-1273 (Moderna)	Day 2 and day 25 after the first dose; day 2 after the second dose	2019	Steroids for 6 months followed by RAASi	No	Yes	0.20	0.34	0.40
	41, F, N/R	BNT162b2 (Pfizer)	Day 2 after the first dose (the patient refused the second dose)	2005	Tac, MPA, and steroids for kidney transplantation	Yes	Yes	0	0.47	0.41
	27, F, N/R	BNT162b2 (Pfizer)	Day 2 after the second dose	2020	Steroid for 1 month followed by RAASi	No	No	20	1.9	1.2
Horino, 2021^11^	46, F, Asian	BNT162b2 (Pfizer)	12 hours after the second dose	4 years before	Prednisolone	Yes	No	N/R	N/R	Nephrotic range
Abramson et al, 2021^20^	30, M, W	mRNA-1273 (Moderna)	Day 2 after the second dose	No history of IgAN	RAASi	At presentation	N/R	N/R	N/R	0.8
Anderegg et al, 2021^21^	39, M, N/R	mRNA-1273 (Moderna)	Immediately after the second dose	No history of IgAN	Cyc + steroids	At presentation	Yes	N/R	N/R	Nephrotic range

^a^Urine microalbumin/creatinine.

Cyc, cyclophosphamide; F, female; M, male; MPA, mycophenolic acid; N/R, not reported; pred, prednisone; RAASi, renin-angiotensin-aldosterone system inhibitor; SARS-CoV-2, severe acute respiratory syndrome coronavirus 2; tac, tacrolimus; W, White.

To our knowledge, only 2 cases of de novo IgA nephropathy after receiving the mRNA-1273 vaccine (Moderna, Inc) have been reported.^20,21^ Our case is unique in a few aspects. First, the diagnosis of IgA nephropathy was de novo after the BNT162b2 (mRNA) Pfizer vaccine. Second, the patient exhibited simultaneous involvement of skin and kidney consistent with IgA vasculitis.

Confirming the diagnosis of IgA vasculitis requires skin or kidney biopsy. Characteristic findings in a skin biopsy, as observed in our patient, include neutrophilic infiltration of the dermal small blood vessel walls associated with fibrinoid necrosis and disruption of the vessel wall.^28^ Kidney biopsy is reserved for patients in whom the diagnosis is uncertain or in cases of persistent hematuria, proteinuria, or decreased kidney function. Arguably, the kidney biopsy was not a necessity in our patient given the high clinical suspicion of IgA vasculitis in the context of the dermopathologic diagnosis of IgA vasculitis. However, because of the lack of reports of similar cases of this nature, because the diagnosis had potential long-term implications, and because the patient was at low risk for procedural complications (normal blood pressure, normal serum creatinine, normal hemoglobin, platelets, and coagulation profile), the decision was made to pursue a kidney biopsy.

## CONCLUSION

Clinicians need to be aware that IgA vasculitis can develop in susceptible individuals following exposure to a COVID-19 vaccination. Careful monitoring of urinalysis and kidney function by primary care providers may be advised, particularly for patients with preexisting glomerular disease (such as IgA nephropathy or minimal change disease) or patients with a history of atopy or seasonal allergies, such as the patient reported in this case.

## Notes

*This article meets the Accreditation Council for Graduate Medical Education and the American Board of Medical Specialties Maintenance of Certification competencies for Patient Care and Medical Knowledge.*
